# Examining Agreement between Clinicians when Assessing Sick Children

**DOI:** 10.1371/journal.pone.0004626

**Published:** 2009-02-27

**Authors:** John Wagai, John Senga, Greg Fegan, Mike English

**Affiliations:** 1 Child and Newborn Health Group, Centre for Geographic Medicine, Kenyan Medical Research Institute (KEMRI)–Wellcome Trust Programme, Nairobi, Kenya; 2 Kenya Medical Research Institute (KEMRI), Centre for Geographic Medicine Research-Coast, Kilifi, Kenya; 3 Infectious Disease Epidemiology Unit, Department of Epidemiology and Population Health, London School of Hygiene and Tropical Medicine, London, United Kingdom; 4 Department of Paediatrics, University of Oxford, Oxford, United Kingdom; Johns Hopkins Bloomberg School of Public Health, United States of America

## Abstract

**Background:**

Case management guidelines use a limited set of clinical features to guide assessment and treatment for common childhood diseases in poor countries. Using video records of clinical signs we assessed agreement among experts and assessed whether Kenyan health workers could identify signs defined by expert consensus.

**Methodology:**

104 videos representing 11 clinical sign categories were presented to experts using a web questionnaire. Proportionate agreement and agreement beyond chance were calculated using kappa and the AC1 statistic. 31 videos were selected and presented to local health workers, 20 for which experts had demonstrated clear agreement and 11 for which experts could not demonstrate agreement.

**Principal Findings:**

Experts reached very high level of chance adjusted agreement for some videos while for a few videos no agreement beyond chance was found. Where experts agreed Kenyan hospital staff of all cadres recognised signs with high mean sensitivity and specificity (sensitivity: 0.897–0.975, specificity: 0.813–0.894); years of experience, gender and hospital had no influence on mean sensitivity or specificity. Local health workers did not agree on videos where experts had low or no agreement. Results of different agreement statistics for multiple observers, the AC1 and Fleiss' kappa, differ across the range of proportionate agreement.

**Conclusion:**

Videos provide a useful means to test agreement amongst geographically diverse groups of health workers. Kenyan health workers are in agreement with experts where clinical signs are clear-cut supporting the potential value of assessment and management guidelines. However, clinical signs are not always clear-cut. Video recordings offer one means to help standardise interpretation of clinical signs.

## Introduction

Improving child survival is a major global health priority with the focus being on low income countries where the largest burden of childhood disease is found. Within these countries pneumonia, diarrhoea, malaria, measles and malnutrition are responsible for up to 80% of infant and child mortality[1]. Case management guidance for these diseases is provided as part of the Integrated Management of Childhood illnesses (*IMCI*) strategy formulated by the WHO and UNICEF. This strategy relies in part on rapid and appropriate recognition of sick children and subsequent prompt treatment and, or referral.

A relatively small set of clinical features are used for the identification and assessment of severity of illness in such approaches. [2] The majority of the recommended clinical features are included on the basis of evidence of their value accumulated over the last thirty years. The research evaluating their utility has largely been based on experts' assessments or assessments made by health workers trained by experts. In the latter case, training is employed to ensure adequate agreement between individuals involved in the research [3], [4], [5], [6], [7], [8], [9]. However, there have been very few attempts to examine whether experts agree [10]. We are also unaware of any specific attempts to explore agreement between experts and health workers in multiple, routine settings receiving no special training for the apparently obvious reason that it is impossible to organise for large numbers of clinicians to observe the same patient at the same time. However, use of video recordings potentially overcomes this problem, at least for selected clinical signs. Video recordings and images have been widely used in developed countries for research, teaching and for assisting patients management. In tele-dermatology for example videos and/or images have been extensively used in competency and agreement studies [11], [12], [13]. Our interest was, therefore, to begin to develop an approach that could explore agreement between experts and identify clear ‘standard’ examples of clinical signs based on their consensus. Once developed identification of these standard signs could be tested amongst health workers in non-research settings who are expected to use guidelines which are based on such clinical signs.

## Methods

### Videos

**Figure 1:**
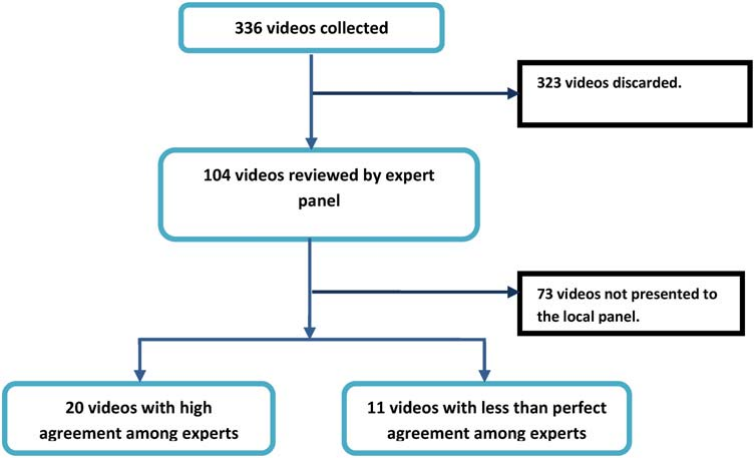
Flow chart representing video selection and presentation to the different panels.

Video recordings of key clinical signs lasting 20–45 seconds were made in children attending Kenyan hospitals after obtaining informed, signed consent from their parents or caretakers and in such a way that treatment was not delayed or interrupted. 336 video recordings of children were made from which 104 videos, displaying one of 11 clinical features (see Table 1), were selected based on the investigators opinion of the quality of the recording. We linked to each clip a single question on the presence or absence of a single clinical sign (or where more appropriate the degree of severity). An international panel of experts (described below) reviewed all videos in January 2008 and 20 videos were identified as clear examples of a child with or without a specific clinical feature after applying criteria of strong consensus. An additional 11 high quality videos were selected for which there was no clear consensus on the presence or absence of one of the clinical features. These 31 video clips then comprised a test set for review by local health workers (see table 2). The process of video selection and presentation to the two panels is demonstrated in figure 1.

**Table 1:** Demographic characteristics of the international (HE) and local panels (hospitals H1 to H8 and the national hospital, HN).

	HE	H 1	H 2	H 3	H 4	H 5	H 6	H 7	H 8	HN
Participants	20	11	15	17	11	21	12	4	8	55^1^
Female [%]	12 [60%]	5 [45%]	8 [53%]	9 [52%]	4 [36%]	10 [47%]	6 [50%]	1 [25%]	4 [50%]	30 [54%]
Number of Interns	0	0	9	12	0	6	9	0	0	0
Median years staff experience [10^th^–90^th^ centile]	14 [7.5–22]	4 [1–14]	3.5 [1–10]	4 [4–4]	4 [1–12]	8.5 [1–28]	1 [1–6]	10 [1–20]	10 [1–25]	6 [4–15]

1 Most of these were consultant paediatricians.

**Table 2:** Expert agreement observed for specific clinical sign groups within the whole panel of examples (n = 104), for signs (n = 20 examples) selected on the basis of very high proportionate agreement (*P*o) and for signs (n = 11) selected where proportionate agreement was low (*no example with low proportionate agreement available).

Clinical category	Options available to panellists	All videos				High consensus set				Low consensus set			
		*N* = 104	*P*o	AC1	*K*appa	*N* = 20	*P*o	AC1	*K*appa	*N* = 11	*P*o	AC1	*K*appa
Acidotic breathing	[Yes] [No]	12	0.68	0.63	0.14	1	1.00	1.00	1.00	2	0.68	0.63	0.14
Capillary refilling	[<2s] [2–3s][>3s]	7	0.73	0.70	0.18	2	1.00	1.00	1.00	2	0.64	0.57	0.11
Sunken eyes	[Yes] [No]	12	0.79	0.77	0.25	2	1.00	1.00	1.00	1	0.47	0.29	0.04
Ability to feed	[Yes] [No]	8	0.84	0.83	0.32	2	1.00	1.00	1.00	1	0.51	0.36	0.05
Indrawing	[Yes] [No]	10	0.72	0.67	0.17	3	0.95	0.95	0.63	1	0.52	0.37	0.06
Head Nodding	[Yes] [No]	9	0.77	0.74	0.22	2	0.95	0.95	0.63	1	0.49	0.32	0.05
Pallor	[0][+][+++]	9	0.71	0.66	0.16	2	0.94	0.95	0.63	1	0.66	0.60	0.12
Skin pinch	[<1s][1–2s][>2s]	14	0.67	0.63	0.14	2	0.96	0.97	0.77	0*	-	-	-
AVPU at Alert	[Yes] [No]	7	0.72	0.68	0.17	2	1.00	1.00	1.00	1	0.52	0.37	0.06
AVPU at Voice	[Yes] [No]	6	0.92	0.92	0.52	1	0.95	0.95	0.63	1	0.53	0.40	0.06
AVPU at Pain	[Yes] [No]	10	0.84	0.83	0.32	1	1.00	1.00	1.00	0*	-	-	-

### International expert panel

Thirty-two leading paediatric clinical researchers and practitioners were approached by email inviting them to review the 104 video clips. Sixteen (16) experts completed the review task using a password protected web questionnaire (http://www.cnhg-kenya.com). Data from the web based questionnaire was collected into a MySQL database available to the authors. Four (4) experts from regions with low internet band-width had to complete the same questionnaire using a memory stick version that generated a simple spreadsheet that could be emailed back to the investigators to give a final expert panel comprising 20 people (listed in the acknowledgements).

### Local panels

The test set of 31 videos were embedded into a Microsoft PowerPoint® presentation. Each slide showed one video and asked the same single question about the presence, absence or grade of a clinical feature that the experts had responded to. Health workers from 8 Kenyan government hospitals in relatively rural districts and from the national hospital viewed the presentation in groups and recorded their opinions on preformatted paper questionnaires. Video viewing continued until all participants had made a decision and without any conferring amongst participants. Health worker panels in district hospitals comprised hospital staff available at the time of the study team's visit who were responsible for administering routine medical care to children. In the national hospital all university consultants and consultant trainees within the university department of paediatrics were invited to participate. In each hospital only one viewing session was offered, taking from 60 to 80 minutes.

### Data processing and Analysis

Responses to clinical sign questions were either dichotomous (present/absent) or one of three ordinal grades (absent or normal/mild-moderate abnormality/severe abnormality). Data were analysed using Stata® version 9.2 (StataCorp, Texas, USA). For all 104 video clips viewed by experts' proportionate agreement, Fleiss's kappa and the AC1 statistic were calculated (The AC1 statistic is designed to correct the overall agreement probability for chance agreement). [14] No weighting was applied. Both the Fleiss's kappa and AC1 statistic generate a number representing the proportionate agreement adjusted for chance where the value 0 means no agreement beyond chance and the value 1 means perfect agreement. We chose to calculate both these statistics as the more commonly used kappa may be misleading as trait prevalence varies [15], [16]. We calculated the AC1 statistic and kappa for individual videos and groups of videos showing the same clinical feature and to examine the difference between AC1 and kappa we plotted the values of both measures for each of the 104 clinical videos reviewed by experts against the underlying proportionate agreement.

To identify the 20 consensus based ‘standard’ video clips we selected those with very high proportionate agreement amongst experts (*P*o≥0.94) on the presence, absence or grade of the sign in question. We also selected 11 video clips of high quality with an absence of consensus (proportionate agreement amongst experts *P*o< = 0.68) to form the 31 video test set. For each local health worker the ability to identify clear-cut clinical signs was assessed by calculating their sensitivity and specificity with respect to the 20 gold-standard video clips. In this analysis sensitivity is interpreted as health workers ability to detect a truly positive clinical sign and specificity as their ability to detect a truly negative clinical sign. Mean sensitivities and specificities were derived for groups aggregated by hospital, clinical background or other characteristics and compared in exploratory analyses. There were no clinically meaningful differences in sensitivities or specificities between groups (data not shown). Agreement statistics, kappa and AC1, were calculated for each hospital group for the 11 videos without a clinical consensus.

### Ethical approval

Taking the videos and the subsequent study were approved by the KEMRI/National Ethical Review Committee.

## Results

Twelve of the 20 international panellists were female, the median years of clinical experience was 14(10^th^–90^th^ percentile: 7.5–22 years). A total of 99 health workers from the district hospitals participated including clinical officer interns, registered clinical officers, medical officer interns, medical officers, nurses, and consultant paediatricians. (A clinical officer is a form of substitute doctor with a 3 year diploma in medicine). The national referral hospital panel included 55 paediatric consultants and registrars (consultant trainees). Details of participants' characteristics are presented in table 1.

**Figure 2:**
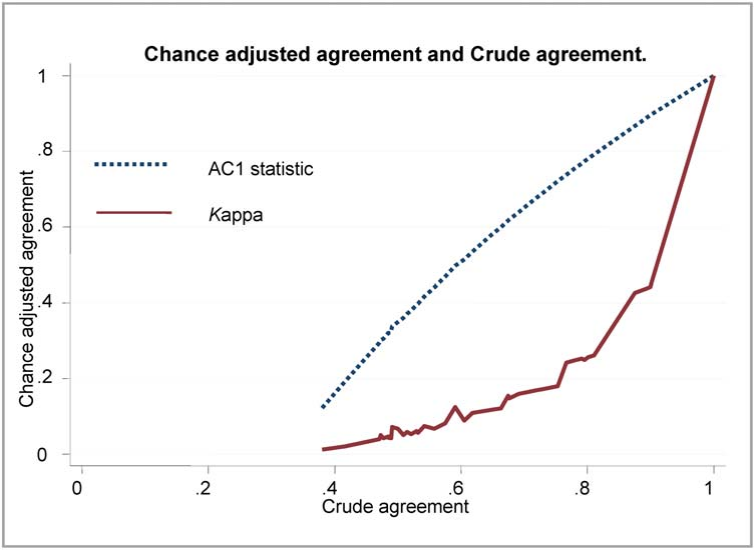
The relationship between AC1 and Kappa statistics to crude agreement unadjusted for chance. The figure demonstrates the relationship between two chance-adjusted measures of agreement the AC1 and kappa statistics and the crude unadjusted agreement represented by the proportionate agreement calculated for responses from a panel of 20 international experts to a single question on a clinical sign for 104 videos.

The AC1 measure of agreement amongst the international experts was generally high for the 104 videos individually ranging from 0.62 to 0.92. For the 20 consensus videos, the AC1 measure of agreement was very high, ranging from 0.95 to 1.00. Agreement as assessed by kappa values was considerably lower than the AC1 in most cases (Table 2) with 29 of the 104 videos associated with poor or fair agreement (kappa<0.4) on a commonly used scale [14]. The relationship between AC1, kappa and the proportionate agreement is demonstrated in Figure 2. In common with the experts agreement scores for the videos in the high consensus set was very good within the 9 Kenyan hospital sites or when analysed according to health worker cadre (data not shown).

Within the high consensus set of videos there was an equal number of videos with either presence or absence of a sign. The local panels achieved high sensitivity across all health worker cadres in identifying the presence of positive clinical signs with sensitivities ranging between 0.927 and 0.975. The specificity across health worker categories was marginally lower at between 0.813 and 0.886 (Table 3). For the 11 videos with no consensus among experts, the crude proportionate agreement (0.48–0.70), AC1 (0.30–0.64) and kappa values (0–0.02) calculated for the different health worker groups or hospitals were low, indicative of absence of agreement in common with the experts.

**Table 3:** Mean sensitivity and specificity of clinicians grouped by their cadre from routine hospital settings in Kenya for identification of the presence or absence of the clinical signs presented within the set of 20 high consensus videos.

Profession	Group size	sensitivity	specificity	*P*o	AC1	***k***appa
CO Intern	30	0.943	0.826	0.790	0.743	0.404
Consultant	15	0.953	0.874	0.821	0.777	0.558
MO	10	0.940	0.894	0.823	0.782	0.412
MO Intern	10	0.927	0.884	0.801	0.758	0.521
RCO	39	0.946	0.813	0.774	0.718	0.332
Registrar	42	0.975	0.886	0.821	0.777	0.558
Nurse	7	0.897	0.823	0.780	0.725	0.586

## Discussion

This study tested a novel method of conducting research on agreement when interpreting clinical signs between expert clinicians who were widely dispersed geographically. The successful use of the internet to host these videos and use of a version contained on a memory stick where internet access is still poor suggests that this approach can be further developed to include clinicians even from remote areas with access to a computer. Such methodologies have obvious extensions to teaching new skills to students and health workers. We then extended the approach, using a group presentation, to explore the ability of health workers in routine practice to identify consensus defined clinical signs.

It is possible that a different set of experts would have classified the signs presented on videos differently. However, we included experts from a wide variety of settings internationally. It is also possible that agreement within local hospital panels was high because we used an open presentation despite our attempts to limit contamination between observers. Despite these potential limitations we believe the study demonstrated that very clear consensus can be reached over the presence (or absence/grade) of specific clinical signs amongst experts. Furthermore it also demonstrated that where experts have a clear view on a clinical sign then health workers of a wide variety of cadres and with widely different levels of clinical experience in routine practice, at least in Kenya, are also able to identify the clinical sign. This provides some reassurance that teaching or guidelines based on these clinical signs have the potential to be understood and implemented widely. However, the study also demonstrated that for many clinical videos experts showed only moderate or even poor agreement. Where experts found it hard to agree health workers in routine settings also found it hard to agree. This finding has several implications.

Firstly, clinical signs may be depicted better as a spectrum from obviously present to obviously not present with the position on the spectrum for any one child or video being best represented by the proportionate agreement amongst multiple, expert observers. The consequence of this is that training people to interpret clinical signs might best be done using videos where possible and a standard set of examples defined by proportionate agreement amongst experts. It will also be clear that any research study or aspect of clinical practice based on clinical sign criteria, whether it is an observational study, a randomised controlled trial or a guideline, will suffer to a greater or lesser degree from misclassification errors as lack of agreement interpreting clinical signs is not uncommon. Standard sets of video records could help improve clinical research and the generalisability of results.

The mean sensitivity scores were marginally higher than the specificity scores. Sensitivity was based on ability of health workers to detect truly positive clinical signs while the specificity was based on the health workers ability to detect truly negative clinical signs. Scoring higher for sensitivity than specificity may be interpreted that the health workers tend to over diagnose; that is any person attending hospital is likely to be labelled as being sick. The clinicians' cautiousness would ensure that sick patients are identified and subsequently treated but the lower specificity may result in overtreatment of children attending hospital who did not need treatment.

When investigating agreement between observers researchers have for a long time used kappa and other chance adjusted measures with a commonly used scale to interpret kappa derived by Landis and Koch in 1977[17]. However, the appropriateness of kappa as a measure of agreement has recently been debated. The dependence of kappa on trait prevalence and on the marginal totals in the cross-tabulation used in its calculation predisposes kappa to two paradoxes. Counter intuitively studies can have high kappa values at relatively low levels of crude agreement and, conversely, there can be low levels of kappa for corresponding high crude agreement [15], [16]. These limitations of kappa mean scores are not comparable across studies and suggests simple scales for their interpretation are unhelpful. A relatively new statistic, the AC1 statistic, has been suggested by Gwet to adjust for chance in agreement studies [14]. In this study we compared crude agreement and chance adjusted agreement using Fleiss' kappa and the AC1 statistic (Figure 2). At the extremes of crude agreement the AC1 and Fleiss' kappa scores approximated each other. For the other values of crude agreement, kappa scores were usually lower than AC1 scores and were not linearly correlated with crude agreement.

In conclusion, we have shown that there can be widespread agreement in identifying obvious examples of clinical signs amongst all types of clinicians. However, greater attention should be paid to establishing where possible standardised thresholds for decisions on when a sign is or is not present, as appropriate, to delineate a particular condition. Video records provide one possible means to achieve this. Clinicians should also be more aware of the development of statistical theory underpinning measures of agreement to avoid well-described pitfalls. This study adds to the wider body of evidence on work done to understand workers abilities in recognising signs recommended by IMCI [3], [4], [5], [6], [7], [8], [9].
